# Stress relaxation constitutive model of rock based on Hausdorff derivative

**DOI:** 10.1371/journal.pone.0346035

**Published:** 2026-04-09

**Authors:** Yuemei Li

**Affiliations:** School of Civil Engineering, Liaodong University, Dandong, China; Tongji University, CHINA

## Abstract

Stress relaxation is a key time-dependent response that governs the long-term stability of deep underground rock engineering. In this study, staged uniaxial and triaxial stress relaxation tests on sandstone were carried out using an MTS815.02 triaxial rheological servo system. Based on the Hausdorff derivative, a fractal-order dashpot was introduced to replace the Newtonian dashpot in the classical Poynting–Thomson model, yielding a nonlinear viscoelastic relaxation model with a time-fractal parameter α. By decomposing stress/strain into spherical and deviatoric parts, the model was further extended to a three-dimensional stress state and verified against triaxial relaxation data. Model parameters were identified by the Levenberg–Marquardt algorithm, and the fitting correlation coefficient for all strain levels exceeded 0.95. A parameter sensitivity analysis quantified the roles of elastic moduli, viscosity, and α in controlling the initial stress level, relaxation magnitude, and relaxation rate. The proposed formulation provides a practical constitutive description for relaxation-dominated deformation of sandstone under high in-situ stress and offers a basis for time-dependent stability assessment and support design in deep rock engineering.

## 1 Introduction

The stress relaxation phenomenon for rock refers to the change of mechanical properties and stress state caused by the change of stress in the rock during loading and unloading [1–2]. This is a significant phenomenon in the field of rock mechanics. It plays a crucial role in influencing the stability, deformation behavior, and engineering design of rock structures.Rock stress relaxation is a prevalent issue in geological engineering [3–4]. When external loads are applied to rock, both elastic strain and plastic strain are induced.When the load is removed, the rock will undergo a certain degree of stress relaxation [5–6]. A phenomenon of stress relaxation is primarily influenced by the rock's inherent physical and mechanical properties. These factors include rock toughness, porosity, permeability, etc [7]. The phenomenon of stress gradually being released over time in rocks makes mechanical properties of rock more complex, which needs to be further studied and understood [8].

In recent decades, rock stress relaxation has been a topic of in-depth exploration and widespread interest among numerous researchers. One of the important research areas is mathematical model of stress relaxation. A constitutive model is a mathematical framework used to represent the mechanical response of rock subjected to various stress conditions. These models help engineers and geologists predict the behavior of rock deformation and failure more accurately. Tian et al. [9] studied the effect of serrated surface inclination on creep and stress relaxation of discontinuous surfaces. The empirical model of shear rheological behavior and the empirical relationship of stress relaxation are additionally formulated. The models presented in this study effectively capture the behavior of shear creep and stress relaxation across different loading paths. Wang et al. [10] established a new variable fractional damage framework to study stress relaxation process of reticulated laterite. The model's analytical results aligned well with the observed data.Compared with other analytical models, the framework could better describe this non-linear characteristics exhibited by the stress relaxation. Zhang et al. [11] researches the shear stress relaxation behavior under varying surface morphologies and early stages of shear stress initiation. They developed both an empirical framework and a nonlinear Maxwell shear stress relaxation framework, which effectively achieved the transition from the deceleration phase of stress relaxation to the stable phase. Zhang et al. [12] developed a relationship between damage variables, time, and model parameters. Incorporating effects of fractional order over time, they proposed a new non-stationary fractional-order constitutive framework for stress relaxation, accounting for aging effects.The test results explored that this framework can effectively describe the complete process of stress relaxation.Rely on the concept of disturbance state, Zhu et al. [13] developed the new stress relaxation framework by connecting spring model and the Merchant model in parallel. The fitted and predicted interfacial stress-relaxation relation curve of rock under axial loading closely adapted to the experimental data. These experimental results confirm applicability and effectiveness of the theoretical model rely on DSC. Yu et al. [14] proposed the damage variable was introduced to account for the weakening of rock parameters. By incorporating this damage variable into the Hooke-Kelvin model, a new nonlinear damage framework was developed.This model was able to accurately describe the extent of damage caused by stress relaxation in rock.

Although some research results have been achieved in the constitutive relationship model for rock stress relaxation.However, there are not many achievements in studying the triaxial constitutive relationship model for rock stress relaxation on the basis of experiments. As a result, it is very necessary to develop rock triaxial stress relaxation test. On this basis, the constitutive model for rock stress relaxation under three-dimensional stress conditions is constructed. At the same time, the commonly used method to improve the model is to use fractional order theory. However, the solution and model effective validation of fractional order are more complicated [15–16]. The applicability of the fractional order calculus theory in rock stress relaxation characteristics still needs to be verified by further theoretical research and experiments [17–18]. As a result, it is essential to develop research on rock stress relaxation model. Finding a rock stress relaxation model with less parameters, high precision and convenient of computational methods is of significant importance for the improvement of rock stress relaxation theory and scientifically evaluating the long-term safety of rock engineering.

In view of this, the present study introduces the Hausdorff derivative into the classical Poynting–Thomson framework by replacing the Newtonian dashpot with a fractal-order dashpot, thereby establishing a nonlinear viscoelastic stress relaxation model for rock. The model is validated through uniaxial and triaxial relaxation tests on sandstone, and the influences of key parameters are clarified via sensitivity analysis. Compared with conventional fractional-order formulations (e.g., Caputo or Riemann–Liouville operators), the Hausdorff derivative is interpreted as differentiation with respect to the fractal time measure tα. This approach retains time-memory effects while yielding an exponential-type analytical solution scaled by tα, which avoids special functions and facilitates numerical implementation. The main contribution of this model lies in its ability to achieve high fitting accuracy with a compact parameter set, where the parameter α directly reflects the heterogeneity of relaxation time scales in rock, offering a constitutive tool that balances physical interpretability with practical usability for stress relaxation analysis.

## 2 Maxwell ‘s body stress relaxation model based on Hausdorff derivative

According to Reference [19], the definition of Hausdorff derivative is


$$\frac{du}{dt^{\alpha }}=\underset{t^{\prime }\to t}{lim}\frac{u\left(t\right)-u\left(t^{\prime }\right)}{t^{\alpha }-{t^{\prime }}^{\alpha }}=\frac{\text{1}}{\alpha t^{\alpha -1}}\frac{du}{dt}$$ (1)

where α is the time-fractal parameter, and *u*(t) is a specific function. Here, 0 < α ≤ 1 denotes the time-fractal parameter. Mathematically, the Hausdorff derivative is equivalent to an ordinary derivative taken with respect to the fractal time variable t^α^. Physically for sandstone, α is a phenomenological descriptor of the distribution of intrinsic relaxation time scales caused by hierarchical microstructures (pores and microcracks), frictional sliding at grain boundaries, and progressive damage evolution. A smaller α indicates stronger temporal heterogeneity and longer memory (slower early-time stress decay), whereas a larger α corresponds to a more homogeneous relaxation process and a faster relaxation rate.

**Fig 1:**
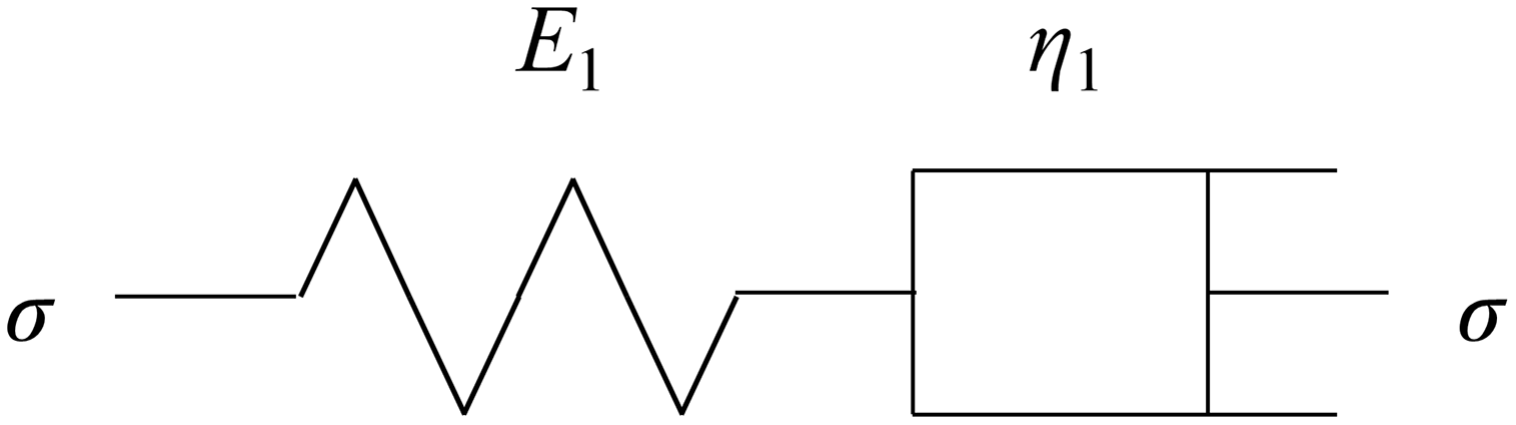
Maxwell ‘s body physical model.

According to the series rule and superposition principle, the Maxwell body composed of spring element and Newton body is obtained as shown in Fig 1 [20].

The rheological equation of the model is


$$\frac{d\varepsilon }{dt}=\frac{d\sigma }{E_{\text{1}}dt}+\frac{\sigma }{\eta_{\text{1}}}$$ (2)

where *E*1 is the stiffness coefficient of the elastic element in the Maxwell body, *η*_1_ is viscosity value of Newtonian fluid in Maxwell ‘s body.

Since the strain ε is a constant in the rock stress relaxation test, the strain rate in Eq. (2) is zero.


$$\frac{d\varepsilon }{dt}=0$$ (3)

Combining the Hausdorff derivative of Eq. (1) in Eqs. (2) and (3), we can obtain


$$\frac{d\sigma }{E_{\text{1}}dt^{\alpha_{\text{1}}}}+\frac{\sigma }{\eta_{\text{1}}}=0$$ (4)

where *α*_1_ is the time-fractal parameter of Maxwell's body.

The initial condition is: when *t* = 0, *σ* = *σ*_0_. The Maxwell body stress relaxation model rely on Hausdorff derivative is calculated by integrating Eq. (4).


$$\sigma =\sigma_{\text{0}}exp\left(-\frac{E_{\text{1}}}{\eta_{\text{1}}}t^{\alpha_{\text{1}}}\right)$$ (5)

where *σ*_0_ is the initial stress.

**Fig 2:**
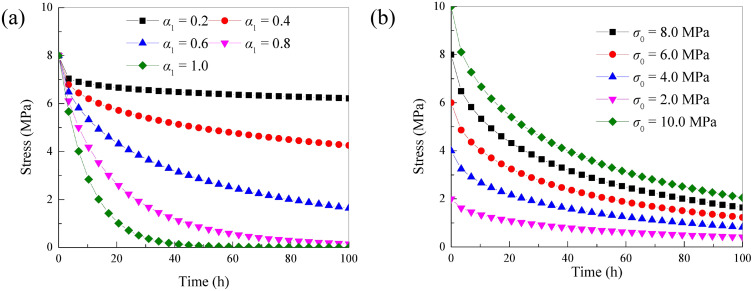
Stress relaxation curves in different situations:(A)different time-fractal parameter *α*_1_. (B)different initial stress value *σ*_0._

When the time-fractal parameter *α*_1_ = 1, the Maxwell stress relaxation framework rely on Hausdorff derivative is reduced to Maxwell stress relaxation framework. The viscosity value of the Newtonian body in the Maxwell body is set to *η*_1_ = 100.00MPa· h. The stiffness coefficient of the elastic element in the Maxwell body is *E*_1_ = 10.00 MPa. The stress relaxation curves under different time-fractal parameter *α*_1_ (initial stress value*σ*_0_ = 8.00MPa) and and initial stress value (time-fractal parameter *α*_1_ = 0.6) are drawn as shown in Fig 2.

**Fig 3:**
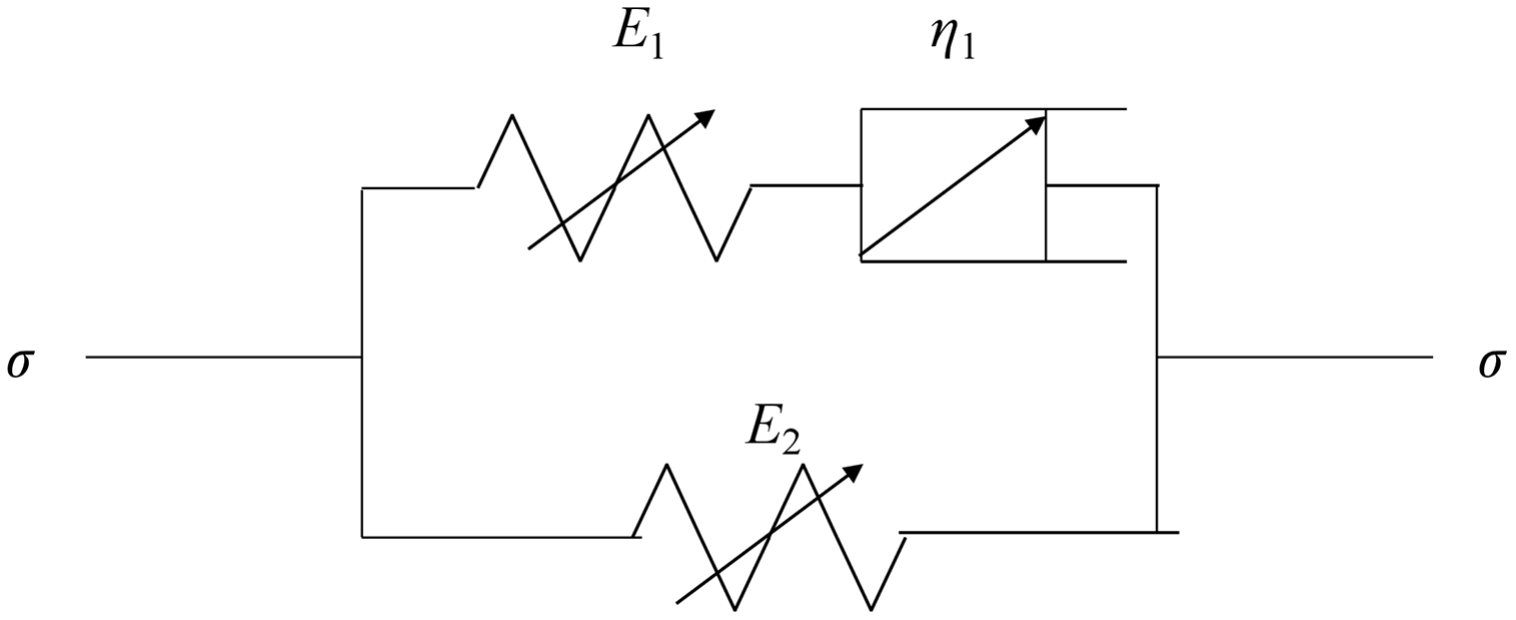
Improved rock stress relaxation model.

As indicated by Fig 2, when the initial stress level is constant and the time-fractal parameter increases, the nonlinear characteristics of the stress relaxation curve become more evident. The decrease in stress and the extent of curve bending also increase. When the time-fractal parameter is constant and the initial stress level increases, the initial point of the stress relaxation curve increases. The nonlinear characteristics of the stress relaxation curve become more evident, and the reduction in stress also increases. To more accurately portray the stress relaxation characteristics of rock under different strain levels, the spring element stress relaxation framework based on the Hausdorff derivative is connected in parallel with the improved Maxwell stress relaxation framework. This improved rock stress relaxation framework is shown in Fig 3. [21].

The rheological equation of framework before improvement is


$$\left(\frac{E_{\text{1}}+E_{\text{2}}}{E_{\text{2}}}\right)\frac{d\varepsilon }{dt}+\frac{E_{\text{1}}}{\eta_{\text{1}}}\varepsilon =\frac{d\sigma }{E_{\text{2}}dt}+\frac{\sigma }{\eta_{\text{1}}}$$ (6)

where *E*_2_ is the stiffness coefficient of the elastic element.

By substituting the value of Eq. (3) into Eq. (6), the improved rock rheological model is achieved by combining Hausdorff derivative of Eq. (1).


$$\frac{E_{\text{1}}}{\eta_{\text{1}}}\varepsilon =\frac{d\sigma }{E_{\text{2}}dt^{\alpha_{\text{2}}}}+\frac{\sigma }{\eta_{\text{1}}}$$ (7)

where *α*_2_ is the time-fractal parameter of the improved rock rheological model.

The initial condition is: when *t* = 0, *σ* = *σ*_0_. The stress relaxation framework rely on Hausdorff derivative is obtained by integrating Eq. (7).


$$\sigma =E_{\text{2}}\varepsilon +\left(\sigma_{\text{0}}-E_{\text{1}}\varepsilon \right)\text{exp}\left(-\frac{E_{\text{1}}t^{\alpha_{\text{2}}}}{\eta_{\text{1}}}\right)$$ (8)

In the field of underground construction, the surrounding rock is often in a multi-directional stress state [22]. However, the traditional one-dimensional stress relaxation framework cannot precisely represent the stress relaxation characteristics of rock under this complex stress state. As a result, it is essential to use the analogy method to expand the stress relaxation constitutive model in three dimensions. Thus, the stress relaxation property of surrounding rock under three-dimensional stress state was better described [23].

Stress tensor of a rock is separated into a spherical stress tensor and a deviatoric stress tensor.Likewise, the strain tensor at any given point within the material can be decomposed into a spherical strain tensor and a deviatoric strain tensor [24].


$$\sigma_{ij}=\sigma_{m}\delta_{ij}+S_{ij}$$ (9)

$$\varepsilon_{ij}=\varepsilon_{m}\delta_{ij}+e_{ij}$$ (10)

where *σ*_*ij*_ is a stress tensor, *σ*_*m*_*δ*_*ij*_ is a spherical stress tensor, *S*_*ij*_ is a deviatoric stress tensor, *ε*_ij_ is a strain tensor at a point in the rock, *ε*_*ij*_*δ*_ij_ is a spherical strain tensor, and *e*_ij_ is a deviatoric strain tensor.

The strain and stress meet this following conditions [25].


$$\left\{ \begin{array}{c} @l@e_{ij}=\frac{\text{1}}{2G}S_{ij}\\ \varepsilon_{kk}=\frac{\text{1}}{3K}\sigma_{kk} \end{array}\right.$$ (11)

where *ε*_*kk*_ is the first invariant of the strain tensor, *σ*_*kk*_ is the first invariant of the stress tensor, *G* is the shear modulus, *K* is the bulk modulus.

Under the triaxial stress state, the total stress *σ* of the improved rock stress relaxation model is


$$\sigma_{11}=\overset{\text{1}}{\underset{11}{\sigma }}+\overset{\text{2}}{\underset{11}{\sigma }}$$ (12)

where *σ*_11_^1^ is stress of the spring element under the triaxial stress state, *σ*_11_^2^ is stress of Maxwell ‘s body under the triaxial stress state.

Rely on generalized Hooke's law and analogy approach, the stress *σ*_11_^1^ of the spring element is


$$\overset{\text{1}}{\underset{11}{\sigma }}=3K_{\text{2}}\varepsilon_{m}\delta_{11}+2G_{\text{2}}e_{11}$$ (13)

where *K*_2_ is the bulk modulus of the spring element, *G*_2_ is the shear modulus of the spring element. Under triaxial compression, rock failure occurs mainly through compressive-shear sliding along failure surfaces. Internal damage, manifested as microcrack initiation and propagation along with pore dilation, primarily influences the shear modulus and viscosity coefficient, with minimal impact on the bulk modulus [26]. Accordingly, the model accounts for damage effects on the shear modulus and viscosity while neglecting its effect on the bulk modulus. Given this behavior, rock volumetric deformation can be treated as elastic, and stress relaxation can be attributed largely to deviatoric strain.

Based on Eq. (8), the improved Maxwell stress-relaxation formulation can be implemented for triaxial stress states by explicitly retaining the time-fractal parameter *α*_2_ as the parameter describing time-memory effects.. Under triaxial confinement, stress relaxation is still governed by the same class of dissipative micro-mechanisms (interface creep, frictional sliding, and defect-related local inelasticity), while confining pressure mainly reshapes their characteristic time spectrum. Therefore, the applicability of the model under triaxial conditions is established on the physical interpretation of α_2_ as a time-memory descriptor, and α_2_ should be identified under the corresponding stress condition rather than assumed invariant.


$$\overset{\text{2}}{\underset{11}{\sigma }}=\left(\sigma_{\text{0}}-2G_{\text{1}}\varepsilon \right)\text{exp}\left(-\frac{G_{\text{1}}t^{\alpha_{\text{2}}}}{\eta_{\text{1}}}\right)$$ (14)

By substituting Eqs. (14) and (15) into Eq. (12), we get


$$\sigma_{11}=3K_{\text{2}}\varepsilon_{m}\delta_{11}+2G_{\text{2}}e_{11}+\left(\sigma_{\text{0}}-2G_{\text{1}}e_{11}\right)\text{exp}\left(-\frac{G_{\text{1}}t^{\alpha_{\text{2}}}}{\eta_{\text{1}}}\right)$$ (15)

In summary, Eq. (15) is an improved rock stress relaxation model based on Hausdorff derivative in three-dimensional state. Physically, increasing confining pressure promotes microcrack closure and enlarges the real contact area at grain and asperity interfaces, which tends to reduce intermittency and narrow the relaxation-time spectrum; consequently, α_2_ may shift toward 1 and the relaxation response becomes closer to a single-time-scale exponential decay. Conversely, under lower confinement, open defects and stronger heterogeneity can enhance long-tail memory, leading to smaller α_2_.

## 3 Rock stress relaxation characteristics test

The equipment used in this test is MTS815.02 rock test system. The rock is taken from the face of a roadway. The rock has been identified as sandstone. Its mineral composition is primarily composed of quartz (approximately 60–65%), feldspar (approximately 20–25%), along with minor amounts of mica and clay minerals. The bulk density of the sample ranges from 2450 to 2500 kg/m^3^, and its porosity is approximately 3.7% to 5.0%. The roadway is buried at a depth of approximately 500 meters. Following the in-situ stress test, the maximum horizontal in-situ stress was found to reach 30 MPa. The vertical ground stress is 15 MPa. Therefore, In this study, 15 MPa was selected as a representative confining pressure because it is close to the measured vertical in-situ stress at the project depth and thus reflects a typical high-stress confinement condition for the investigated sandstone. According to recommended method of the test procedure of the International Society of Rock Mechanics, the rock sample was shaped into a standard cylindrical form with a height of 100 mm and a diameter of 50 mm.The flatness of the end face is maintained within 0.02 mm, while the diameter deviation is kept within 0.30 mm. The samples with cracks visible to the naked eye were removed. Finally, the wave velocity of the rock sample is measured by the wave velocity instrument, and the rock sample with similar wave velocity is taken as the sample object. This reduces discrete difference between rock samples.

### 3.1 Rock mechanical properties test

The test steps of triaxial compression test are as follows [27–28].

(1) A waterproof heat shrinkable film is wrapped around the outer layer of the rock sample. The heating equipment is used to make the thermoplastic film in close contact with the sample.
(2) The axial displacement sensor and the chain lateral displacement sensor are installed outside the above sample combination. And ensure that the sensor and the sample section maintain a horizontal relationship.
(3) Placing the sample combination on the test loading device. And it is essential to carefully connect the sensor loading equipment. Slowly drop the test loading chamber. And it is essential to ensure that the connecting line of the test measurement system completely enters the cabin;
(4) Axial stress is set to some specified value at a loading rate of 0.002 mm/s. And maintaining a constant confining pressure throughout the testing process is essential. At the same time,To prevent sudden brittle failure of the rock, an upper safety limit for displacement must be established before applying axial stress. The confining pressure was first increased to the target value and then held constant; after stabilization (pressure fluctuation less than 0.1 MPa for approximately 10 min), axial loading was applied.
(5) Axial stress is applied to the specimen at a rate of 0.002 mm per second. Once rock reaches peak strength failure, axial displacement at the same rate is continuously applied until the residual strain is measured.
(6) It is essential to save the measured data.Circumferential pressure must be unloaded first, followed by the axial stress.
(7) After extracting the hydraulic oil from the chamber, the damaged rock samples are removed, labeled, and stored.
(8) According to the test measurement data, stress-strain relation curve of rock under axial loading is drawn.

**Fig 4:**
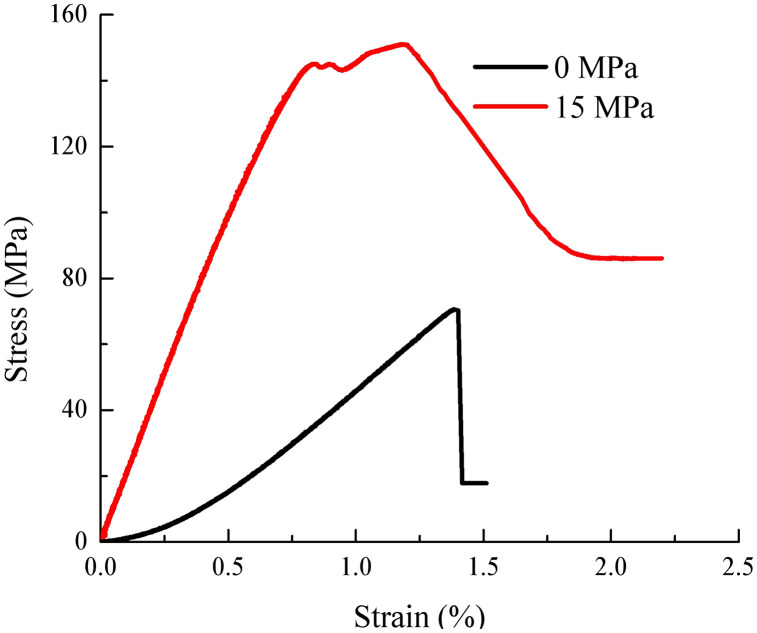
Axial stress-strain curves of rocks.

Stress-strain relation curve of rock under axial loading is plotted as indicated by Fig 4.

As indicated by Fig 4 that during the pre-peak deformation stage, the stress in the rock increases as the strain grows, continuing until the peak strength is reached. In the post-peak deformation stage, the stress in the rock decreases as the strain increases, eventually reaching the residual strength.During the residual deformation stage, the stress in the rock remains constant as the strain continues to increase.

### 3.2 Rock stress relaxation characteristics test

The test steps of triaxial stress relaxation test are as follows [29].

(1) Circumferential pressure is set to some specified value at a loading rate of 0.002 mm/s. And maintaining a constant confining pressure throughout the testing process is essential.
(2) The maximum axial strain (peak strain)that stage before the triaxial compression test reaches the peak value is measured, which is used as the classification basis. The axial compression was loaded by strain control, and the axial deformation loading rate is a rate of 0.002 mm per second.
(3) Based on the concept of gradient damage, the stable crack propagation stage was selected as the starting point for stress relaxation, allowing the relaxation response to be measured under a repeatable damage state. The strain in the first stage was set to 0.20%, which lies within the pre‑peak deformation range. Each subsequent stage was increased by 20% relative to the preceding one, covering the elastic, yield, peak, softening, and residual stages, while avoiding excessive loading steps and cumulative disturbances.
(4) It is essential to maintain stable strain levels at each stage. When the stress relaxation rate is slower than 0.001 MPa / s, it is deemed that stress relaxation reaches a stable stage. The next level of stress relaxation can be carried out, and this operation can be repeated until the rock sample is destroyed.
(5) Once the hydraulic oil has been removed from the tank, the damaged rock samples are taken out, marked and stored properly.

**Fig 5:**
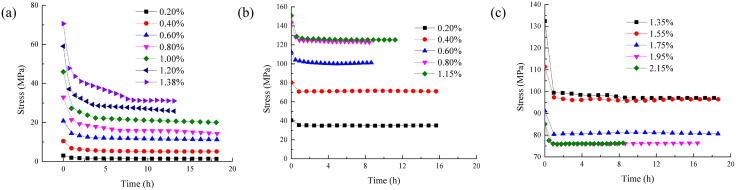
Stress relaxation test curves.

Stress-strain relation curve of rock under axial loading is drawn as indicated by Fig 5.

As shown in Fig 5 that similar to the pre-peak, the post-peak rock also has obvious stress relaxation. Stress-strain relation curve under axial loading of the samples at pre-peak and post-peak strain levels are obviously divided into two different stages. These two stages include fast relaxation stage and deceleration relaxation stage.

Rapid relaxation stage: stress decreases rapidly in a short time after keeping the strain constant.

Deceleration relaxation stage: As time progresses, the stress relaxation rate gradually slows down. The stress approaches a stable value. Compared with the rapid relaxation phase prior to peak stress of rock under the first-order strain after the peak is more obvious. That is, the instantaneous stress reduction characteristics of rock are significant. However, the characteristics of the deceleration relaxation stage of the post-peak rock are relatively not obvious. However, the rapid relaxation stage of the rock under the action of the subsequent strain grade is basically the same as the rapid relaxation stage of the pre-peak rock.

## 4 Verification of rock stress relaxation model

### 4.1 Parameter-strain functional relationships

According to Eq. (8), Levenberg-Marquardt algorithm [30] is employed to determine this test curve in Fig. 5, while this parameters of improved rock sstress relaxation framework, incorporating the Hausdorff derivative in the one-dimensional state for both pre-peak and post-peak samples across various strain levels, are presented in Table 1.

**Table 1:** Parameters of improved rock stress relaxation model.

Strain (%)	*E*_2_ (MPa)	*E*_1_ (MPa)	*η*_1_ (MPa·h)	*α*_2_	*R* ^2^
0.20	683.627	683.820	610.097	0.457	0.985
0.40	1263.970	1264.415	1138.308	0.508	0.979
0.60	1867.987	1867.415	1623.775	0.456	0.994
0.80	1707.526	1707.110	1819.377	0.383	0.995
1.00	1972.090	1972.041	1571.293	0.385	0.989
1.20	2092.133	2092.030	1745.068	0.402	0.990
1.38	693.157	694.246	1334.835	0.301	0.995

As can be seen from Table 1, With increasing strain, model parameters (e.g., *E*_2_,α_2_) vary significantly, driven by the progressive damage evolution in sandstone—namely, microcrack propagation and pore expansion. This microstructural alteration further modulates the rock's elastic–viscoelastic response. To address the resulting parameter instability across strain levels, quadratic regressions were fitted using pre-peak data (ε = 0.20–1.20%; ε in %, parameters in units per Table 1):


$$\begin{array}{c} E_{\text{2}}\left(\varepsilon \right)=-1633.62\varepsilon^{\text{2}}+3573.70\varepsilon +87.36,R^{\text{2}}=0.937\\ \eta_{\text{1}}\left(\varepsilon \right)=-2101.06\varepsilon^{\text{2}}+3965.69\varepsilon -83.35,R^{\text{2}}=0.943 \end{array}$$ (16)

Because *E*_1_ ≈ *E*_2_ at all strain levels, *E*_1_ (ε) can be taken as *E*_2_(ε) for implementation without noticeable loss of accuracy. For the time-fractal parameter, a quadratic fit over ε = 0.20–1.20% yields:


$$\alpha_{\text{2}}\left(\varepsilon \right)=0.3804\varepsilon^{\text{2}}-0.7501\varepsilon +0.753,R^{\text{2}}=0.965$$ (17)

Substituting these relationships into Eqs. (8) and (15), the model can predict stress relaxation behavior under unknown strain levels without re-fitting. Post-peak point (ε = 1.38%) shows a significant decrease in *E*_1_ and *E*_2_, indicating a phase transition dominated by macro-crack coalescence; therefore, the analysis of parameter-strain functional relationships is no longer conducted here.

### 4.2 Model-experimental curve fitting analysis

**Fig 6:**
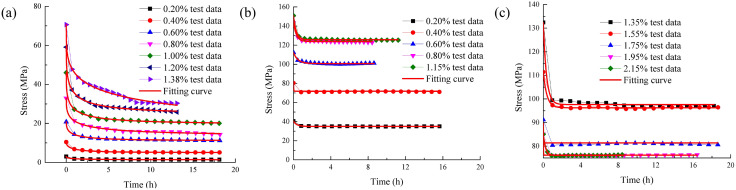
Stress relaxation test curve and model curve.

This parameters of improved rock stress relaxation framework from Table 1 are inserted into Eq. (8), resulting in the stress relaxation test curve and model curve, which are displayed in Fig 6.

As shown in Fig 6 that the improved rock stress relaxation framework based on Hausdorff derivative has the highest fitting accuracy. The fitting correlation coefficient is above 0.95. The model fitting curve almost coincides with the experimental value. From the above discussion, it can be explored that the improved rock stress relaxation framework based on Hausdorff derivative can describe the stress relaxation characteristics of sandstone with greater accuracy.At the same time, the model not only has high precision but also has clear physical meaning.

**Fig 7:**
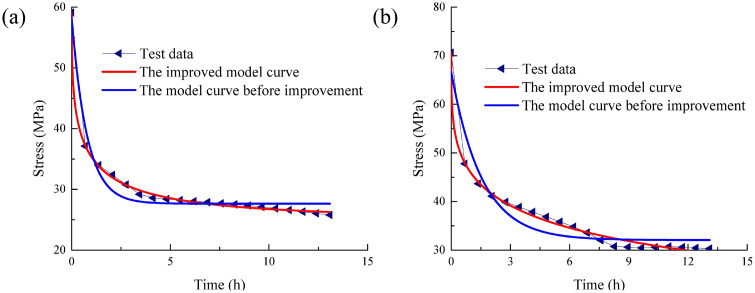
Comparison of rock stress relaxation model curve and test curve before and after improvement:(A)Uniaxial, strain 1.20%. (B) Uniaxial, strain 1.38%.

To further confirm the advantages of the model the rock stress relaxation framework before and after improvement is used to compare the test curves. Fig 7 presents a comparison of the rock stress relaxation framework curve and test framework, highlighting the differences before and after the improvements.

As indicated by Fig.7 that the fitting accuracy of the rock stress relaxation framework before improvement is less than that of the rock stress relaxation framework before improvement. Although correlation coefficient during the fitting process of the rock stress relaxation model before improvement is also greater than 0.90, there is still a certain deviation between the regression curve of the model before improvement and the experimental value. In particular, the deviation at the initial and inflection points is large. In addition, the fitting effect of rock stress relaxation model before improvement is worse under high strain displacement level.

## 5 Sensitivity analysis of model parameters

### 5.1 The influence of parameter *E*_2_

**Fig 8:**
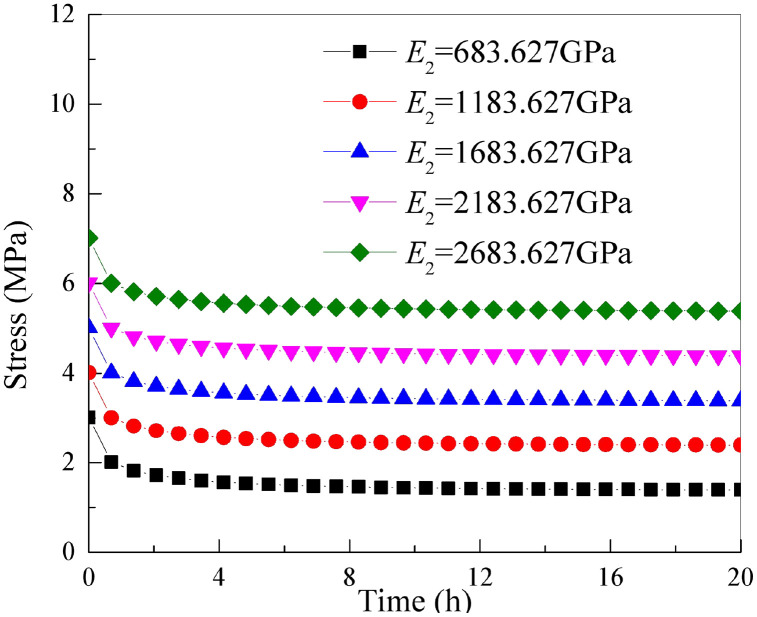
Stress relaxation curves of rock under different parameters *E*_2._

It is essential to keep the other parameters of the improved rock stress relaxation framework rely on the Hausdorff derivative in Table 1 unchanged. By only changing the parameter *E*_2_, the stress relaxation curve of the rock under the action of different parameter *E*_2_ can be obtained as shown in Fig 8.

As shown in Fig 8 that as the parameter *E*_2_ continues to increase, the trend of rock stress gradually decreasing over time remains unchanged. Only the stress level in the early stage of stress relaxation increases..This reveals that the parameter *E*_2_ controls the initial value of the stress relaxation curve.

### 5.2 The influence of parameter *E*_1_

**Fig 9:**
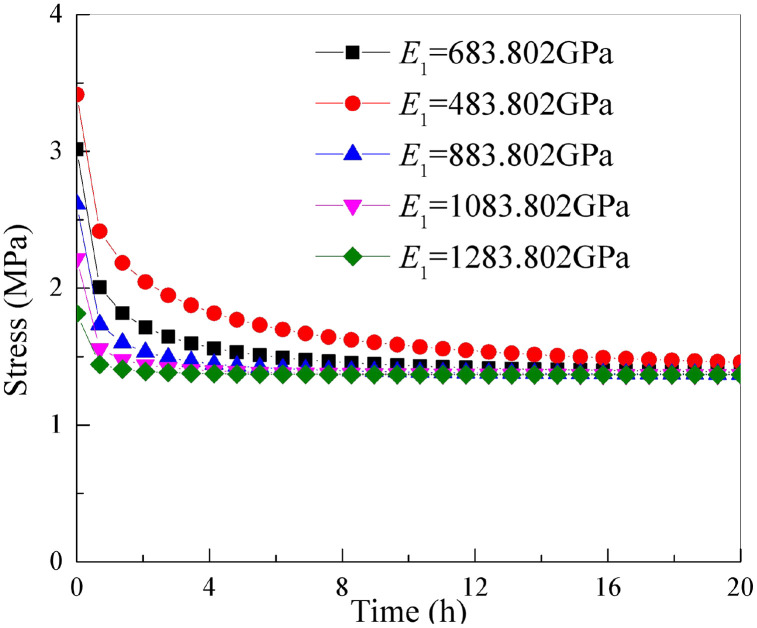
Stress relaxation curves of rock under different parameters *E*_1._

It is essential to keep the other parameters of the improved rock stress relaxation framework rely on the Hausdorff derivative in Table 1 unchanged. By only changing the parameter *E*_1_, stress-relaxation relation curve of rock under axial loading of different parameter *E*_1_ can be obtained as shown in Fig 9.

From Fig 9, it can be seen that with the continuous decrease of the parameter *E*_1_, the greater stress relaxation of the rock (that is, the greater magnitude of stress reduction) and the greater the starting value of stress relaxation curve of the rock. However, as parameter *E*_*1*_ and time progresses, stress-relaxation relation curve of rock is more and more fitted. This shows that the parameter *E*_1_ controls experimental values in the early stage of stress relaxation and degree of stress relaxation.

### 5.3 The influence of parameter *η*_1_

**Fig 10:**
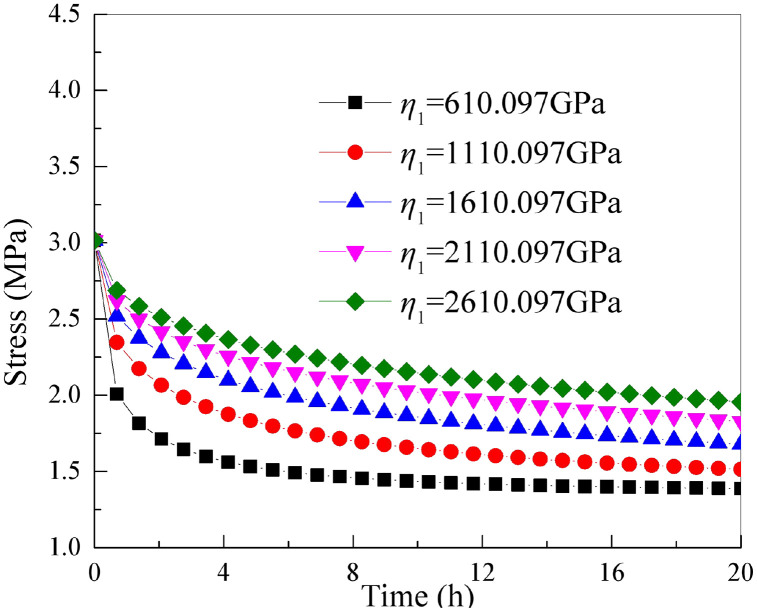
Stress relaxation curves of rock under different parameters *η*_1_.

It is essential to keep the other parameters of the improved rock stress relaxation framework rely on the Hausdorff derivative in Table 1 unchanged. By only changing the parameter *η*_1_, stress-relaxation relation curve of rock under axial loading of different parameter *η*_1_ can be obtained as shown in Fig 10.

From Fig 10, this stress relation curve of rock under different viscosity value is similar. With the continuous increase of parameter *η*_1_, the stress relation of rock decreases (that is, magnitude of stress relaxation decreases). The experimental values in the early stage of stress relaxation is the same. This reveals that the parameter *η*_1_ controls the stress relaxation amount of stress relation curve.

### 5.4 The influence of parameter *α*_2_

**Fig 11:**
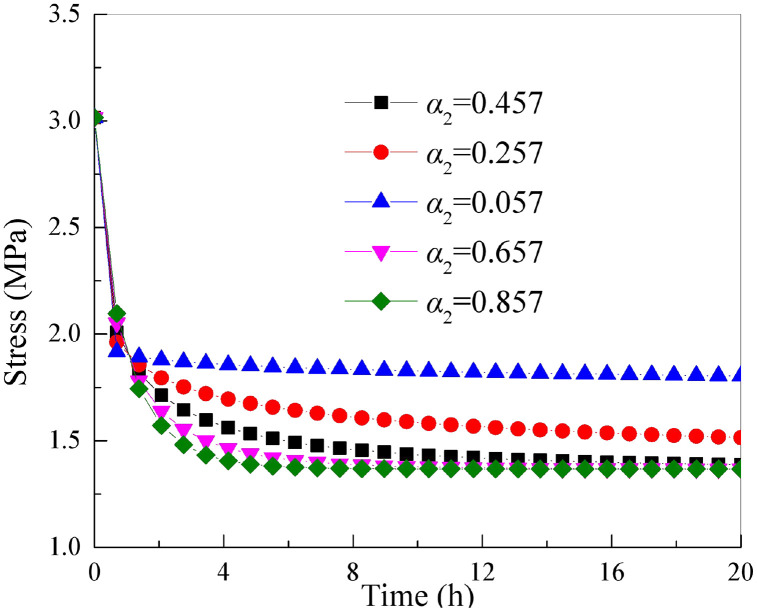
Stress relaxation curves of rock under different parameters *α*_2._

It is essential to keep the other parameters of the improved rock stress relaxation framework rely on the Hausdorff derivative in Table 1 unchanged. By only changing the parameter *α*_2_, stress-relaxation relation curve of rock under axial loading of different parameter *α*_2_ can be obtained as shown in Fig 11.

As shown in Fig 11 that as the parameter *α*_2_ continues to increase, the stress relaxation magnitude of the rock increases (that is, the amount of stress reduction increases), and the stress relaxation rate increases. This shows that the parameter *α*_2_ controls the stress relaxation amount and stress relaxation rate of the stress relaxation curve. Parameter α essentially characterizes the dispersion of the internal relaxation-time spectrum and the strength of the time-memory effect in rock. Mineral composition, porosity, and density can influence the value of α by affecting the compactness of the skeleton, the particle contact state, and the degree of initial defect development. In general, higher density, lower porosity, and a denser quartz–feldspar framework correspond to weaker temporal heterogeneity and thus a relatively larger α value; in contrast, when pores and defects are more developed and the internal heterogeneity is stronger, α may decrease. The sandstone used in this study generally exhibits low porosity, relatively high density, and a compact structure dominated by a quartz–feldspar framework. Therefore, its initial *α*_2_ remains at a moderately high level, indicating that the relaxation process is not extremely discrete. As the strain increases, microcrack propagation and pore expansion gradually intensify, and the originally dense skeleton is continuously disturbed, leading to a renewed broadening of the internal relaxation time-scale distribution and thus causing the staged variation of α_2_. In other words, the mineral composition, density, and porosity mainly determine the initial microstructural basis of α, whereas the damage evolution during loading governs the dynamic adjustment of α_2_.

## 6 Conclusions

A nonlinear viscoelastic stress-relaxation constitutive model was established by introducing the Hausdorff derivative into the classical Poynting–Thomson framework, and was further extended to a three-dimensional stress state. The model captures the staged relaxation behavior of sandstone under uniaxial and triaxial loading, with fitting correlation coefficients higher than 0.95 for all tested strain levels.

Sensitivity analysis shows that *E*_2_ mainly controls the initial stress level at the onset of relaxation, *E*_1_ affects both the early-stage response and the overall magnitude of stress relaxation, *η*_1_ governs the relaxation amount, and α_2_ characterizes the relaxation rate and time-memory effect of the relaxation curve.

The proposed formulation can be used for time-dependent stability assessment and support design in deep rock engineering where stress relaxation contributes to deformation. Nevertheless, because the present triaxial validation was conducted at a representative confining pressure of 15 MPa, further systematic stress-relaxation tests under multiple confining pressures are still needed to quantify the dependence of α_2_ on confinement and to evaluate the model adaptability under broader three-dimensional stress states.

## Supporting information

S1 File Appendix A. (DOCX)
