# Supplementary material for: Stress relaxation constitutive model of rock based on Hausdorff derivative
Source: PLoS One. 2026 Apr 9;21(4):e0346035. doi: 10.1371/journal.pone.0346035 (PMC13065016; doi:10.1371/journal.pone.0346035)
Supplement: S1 File — (DOCX) [file pone.0346035.s001.docx]

**Appendix A. Key derivation steps for Eqs. (5) and (8)**

For the Maxwell element, Eq. (2) gives

In a stress‑relaxation test the strain is held constant, i.e., $\dot{\text{ε}}$=0 which leads to:

Replacing the ordinary time derivative with the Hausdorff derivative yields:

$$\frac{d\sigma}{dt^{\alpha}}=-\frac{E_{1}}{\eta_{1}}\sigma$$

Separating variables and integrating under the initial condition σ(0)=σ_0_ gives:

$$\int_{\sigma_{0}}^{\sigma\left(t\right)} \frac{d\sigma}{\sigma}=-\frac{E_{1}}{\eta_{1}}\int_{0}^{t} d\left(\xi^{\alpha_{1}}\right)$$

which results in:

$$\text{ln}\left[\frac{\sigma\left(t\right)}{\sigma_{0}}\right]=-\frac{E_{1}}{\eta_{1}}t^{\alpha_{1}}$$

and finally:

$\sigma\left(t\right)=\sigma_{0}\exp\left[-\frac{E_{1}}{\eta_{1}}t^{\alpha_{1}}\right]$ （Eq.5）

For the improved model (a spring E2 in parallel with the Maxwell element), the total stress is:

$$\sigma\left(t\right)=E_{2}\varepsilon+\sigma_{M}\left(t\right)$$

Where σ_M_(t) denotes the stress in the Maxwell branch. Under the same constant‑strain condition, σ_M_(t) satisfies the same Hausdorff‑type evolution equation. Applying the initial condition σ(0)=σ_0_ gives:

$$\sigma_{M}\left(t\right)=\left(\sigma_{0}-E_{2}\varepsilon\right)\exp\left[-\frac{E_{1}}{\eta_{1}}t^{\alpha_{2}}\right]$$

Substituting σ*_M_*(t) into the total‑stress expression leads to:

$\sigma\left(t\right)=E_{2}\varepsilon+\left(\sigma_{0}-E_{2}\varepsilon\right)\exp\left[-\frac{E_{1}}{\eta_{1}}t^{\alpha_{2}}\right]$ （Eq.8）

These derivations provide the closed‑form solutions used in the proposed stress‑relaxation constitutive model.
